# Ca^2+^/Calmodulin-Dependent Protein Kinase II (CaMKII) Activity and Sinoatrial Nodal Pacemaker Cell Energetics

**DOI:** 10.1371/journal.pone.0057079

**Published:** 2013-02-25

**Authors:** Yael Yaniv, Harold A. Spurgeon, Bruce D. Ziman, Edward G. Lakatta

**Affiliations:** Laboratory of Cardiovascular Science, Gerontology Research Center, Intramural Research Program, National Institute on Aging, National Institutes of Health, Baltimore, Maryland, United States of America; University of Milan, Italy

## Abstract

Ca^2+^-activated basal adenylate cyclase (AC) in rabbit sinoatrial node cells (SANC) guarantees, via basal cAMP/PKA-calmodulin/CaMKII-dependent protein phosphorylation, the occurrence of rhythmic, sarcoplasmic-reticulum generated, sub-membrane Ca^2+^ releases that prompt rhythmic, spontaneous action potentials (APs). This high-throughput signaling consumes ATP.

**Aims:**

We have previously demonstrated that basal AC-cAMP/PKA signaling directly, and Ca^2+^ indirectly, regulate mitochondrial ATP production. While, clearly, Ca^2+^-calmodulin-CaMKII activity regulates ATP consumption, whether it has a role in the control of ATP production is unknown.

**Methods and Results:**

We superfused single, isolated rabbit SANC at 37°C with physiological saline containing CaMKII inhibitors, (KN-93 or autocamtide-2 Related Inhibitory Peptide (AIP)), or a calmodulin inhibitor (W-7) and measured cytosolic Ca^2+^, flavoprotein fluorescence and spontaneous AP firing rate. We measured cAMP, ATP and O_2_ consumption in cell suspensions. Graded reductions in basal CaMKII activity by KN-93 (0.5–3 µmol/L) or AIP (2–10 µmol/L) markedly slow the kinetics of intracellular Ca^2+^ cycling, decrease the spontaneous AP firing rate, decrease cAMP, and reduce O_2_ consumption and flavoprotein fluorescence. In this context of graded reductions in ATP demand, however, ATP also becomes depleted, indicating reduced ATP production.

**Conclusions:**

CaMKII signaling, a crucial element of normal automaticity in rabbit SANC, is also involved in SANC bioenergetics.

## Introduction

The rate at which the heart beats is governed by the rate at which sinoatrial node cells (SANC) fire spontaneous action potentials (APs). Experimental and computational data (cf [1] for review) support the idea that spontaneous AP generation in mammalian SANC is regulated by a coupled-clock function, i.e. surface membrane electrogenic proteins, functioning as a voltage oscillator (“Membrane clock”), and sarcoplasmic reticulum function as an intracellular generating rhythmic Ca^2+^ oscillator (“Ca^2+^ clock”).

Both, cAMP-mediated, protein kinase A-dependent (PKA) protein phosphorylation and Ca^2+^/calmodulin-dependent protein kinase II (CaMKII) protein phosphorylation (phospholamban, ryanodine-receptors, L-type channel and etc.) couple the function of proteins of both clocks to regulate SANC normal automaticity [1], [2]. It has been demonstrated in sinoatrial node cells that Ca^2+^ activated adenylyl cyclase produces a high basal level of cAMP compared to ventricular myocytes [3], [4]. Adenylate cyclase (AC) activity within lipid microdomains is activated by Ca^2+^ over the entire physiological Ca^2+^ range. Specifically, a reduction in intracellular Ca^2+^ by BAPTA reduced the cAMP level [3]. The level of Ca^2+^ pumping by SR Ca^2+^-ATPase is regulated by phospholamban phosporylation of both Ser^16^ (PKA) and Thr^17^ (CaMKII) [5]. It was shown that the level of phospholamban phosporylation in SANC is associated with the SR refilling rate [6]. Moreover, a decrease in CaMKII results in a decrease of L-type Ca^2+^ current amplitude and a reduction in Ca^2+^ influx [7], [8] that can lead to a decrease in cytosolic Ca^2+^ and a decrease in the availability of Ca^2+^ for pumping into the SR. A reduction in cytosolic Ca^2+^ which leads to a reduction of Ca^2+^ activation of adenylate cyclase (AC), therefore, reduces cAMP activation of PKA, reduces phospholamban phosporylation and Ca^2+^ cycling kinetics. We have recently shown that this feed-forward basal Ca^2+^-cAMP/PKA signaling that drives spontaneous APs, not only regulates ATP consumption of SANC, but also regulates mitochondrial ATP production [9]. For example, the intracellular Ca^2+^ chelator, BAPTA, not only blocks Ca^2+^-dependent activation of CaMKII and suppresses AC/PKA signaling, but also reduces ATP in the context of a reduced ATP demand [9]. We hypothesized that basal state calmodulin-CaMKII signaling is not only required to drive spontaneous APs in rabbit SANC (because CaMKII inhibitors suppress SANC pacemaking [7] and on this basis is linked to ATP utilization), but is also coupled to ATP production.

## Results

**Figure 1:**
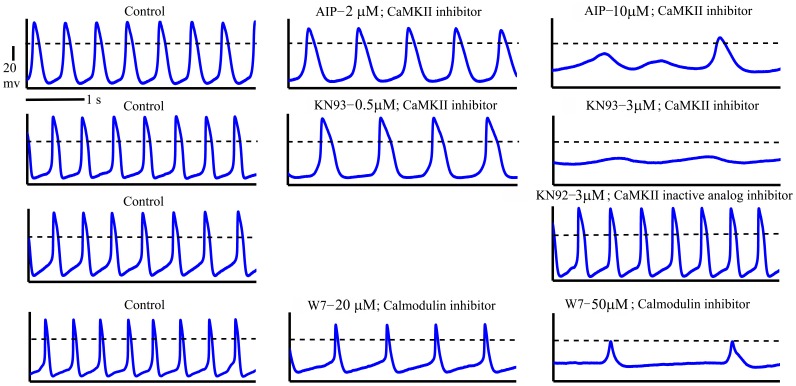
Representative examples of change in spontaneous AP in response to decrease in CaMKII activity or calmodulin.

**Figure 2:**
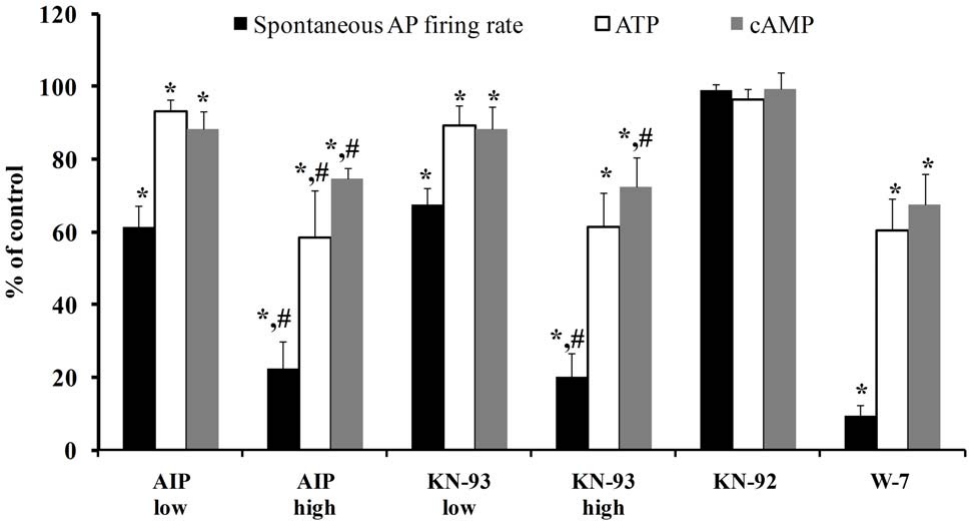
Average changes in (A) spontaneous AP firing rate, (B) ATP and (C) cAMP by decreasing CaMKII activity with KN-93 (high-3 µmol/L, low-0.5 µmol/L), AIP (high-10 µmol/L, low-2 µmol/L), or decreasing calmodulin activity by W-7 (high-50 µmol/L, low-20 µmol/L). *p<0.05 vs. drug control. ^#^p<0.05 vs. drug low concentration.

To decrease CaMKII activity we chose two concentrations of CaMKII inhibitors that had been previously shown [7] to reduce the AP firing in rabbit SANC rate by ∼40%, and to eliminate AP firing. Graded reductions in basal CaMKII activity by application of CaMKII inhibitors for 5 min (AIP 2 µM or KN-93 0.5 µM compared to AIP 10 µM or KN-93 3 µM) result in graded reductions in the spontaneous AP firing rate of single SANC (see representative examples on Fig. 1, Fig. 2). On average, 2 µM AIP reduced the spontaneous AP firing by 39±6%, while 0.5 µM KN-93 reduced it by 33±5%; 10 µM AIP reduced the spontaneous AP firing by 77±7%, while 3 µM KN-93 reduced it by 80±6%. In contrast, 3 µmol/L KN-92, a structural analog of KN-93 that does not inhibit CaMKII activity, did not significantly change the AP firing rate (on average the spontaneous AP firing was reduced by only 1±2%) (Fig. 2). After 5-min, the steady-state effects of the CaMKII inhibitors on AP firing rate achieved were similar to their effects documented previously [7]. The effect of KN-93 on the AP firing rate was not reversible (after 10 min washout with Tyrode solution), but the AIP effect was partially reversible (for more details see [7]).

Concentrations of the calmodulin inhibitor, W-7, were chosen to match the reduction in AP firing rate achieved with inhibition of CaMKII activity. Graded concentrations of W-7 (20 to 50 µM) for 5 min, also produced graded reductions in the spontaneous AP firing rate (Fig. 2). On average, 20 µM W-7 reduced the spontaneous AP firing by 42±8%, while 50 µM W-7 reduced it by 90±3%. The steady-state effect of W-7 on AP firing was achieved after 5 min. The effect of W-7 on the AP firing rate was not reversible.

**Figure 3:**
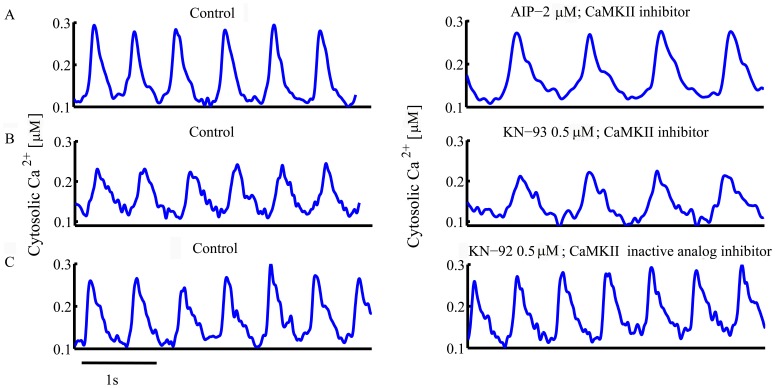
Representative examples of change in Ca^2+^ transient in response to decrease in CaMKII activity or calmodulin.

Fig. 3 shows the Ca^2+^ transients measured with indo-1 during partial inhibition of CaMKII or calmodulin (AIP 2 µM, KN-93 0.5 µM or W-7 20 µM). CaMKII or calmodulin inhibitors also markedly slow the kinetics of Ca^2+^ removal from the cytosol in single SANC (Tables 1 and 2), which, in part, reflects a change in intracellular Ca^2+^ cycling, i.e., a reduction of cytosolic Ca^2+^ uptake by the SR [10] and of SR Ca^2+^ release by ryanodine receptors [2], and a reduction in L-type current [7], [11].

**Table 1:** Average cells AP induced Ca^2+^ parameters in control (CON) and following introduction of AIP (n = 6; 2 µmol/L), KN-93 (n = 6; 0.5 µmol/L) or KN-92(n = 5; 3 µmol/L), *p<0.05 vs. drug control.

	CON	AIP	% Change	CON	KN-93	% Change	CON	KN-92	% Change
**Peak Systolic Ca^2+^** **(nmol/L)**	305±11	272±10^*^	−11±1^*^	295±9	264±14^*^	−10±4^*^	304±7	308±7	2±2
**Minimum diastolic** **Ca^2+^ (nmol/L)**	101±12	100±12	−1±3	111±9	106±9	−4±3	104±10	109±11	4±3
**Ca^2+^ amplitude** **(systolic-diastolic)** **(nmol/L)**	205±13	171±10^*^	−16±3^*^	184±7	158±11^*^	−13±5^*^	200±10	199±5	−1±3
**T-P_c_ (ms)**	80±4	85±5	7±3	80±6	88±8	10±3	76±9	80±10	5±4
**T-50_c_ (ms)**	176±15	205±17^*^	16±3^*^	172±7	199±7^*^	16±4^*^	188±13	200±1	12±8
**T-90_c_ (ms)**	302±9	338±11^*^	12±2^*^	305±11	323±13^*^	8±4^*^	273±19	270±18	−2±3
**Beats per min**	130±6	79±6^*^	−39±6^*^	141±2	96±7^*^	−33±5^*^	130±9	129±10	−1±2

**Table 2:** Average cells AP induced Ca^2+^ parameters in control (CON) and following introduction of W-7 (20 µmol/L; n = 6), *p<0.05 vs. drug control.

	CON	W-7	% Change
**Peak Systolic Ca^2+^** **(nmol/L)**	277±9	242±6^*^	−12±4^*^
**Minimum diastolic** **Ca^2+^ (nmol/L)**	102±14	96±13	−4±7
**Ca^2+^ amplitude** **(systolic-diastolic)** **(nmol/L)**	172±15	146±16^*^	−17±8^*^
**T-P_c_ (ms)**	80±5	82±4	2±1
**T-50_c_ (ms)**	188±11	213±17^*^	13±4^*^
**T-90_c_ (ms)**	286±18	340±15^*^	18±7^*^
**Beats per min**	144±9	83±9^*^	−42±8^*^

**Figure 4:**
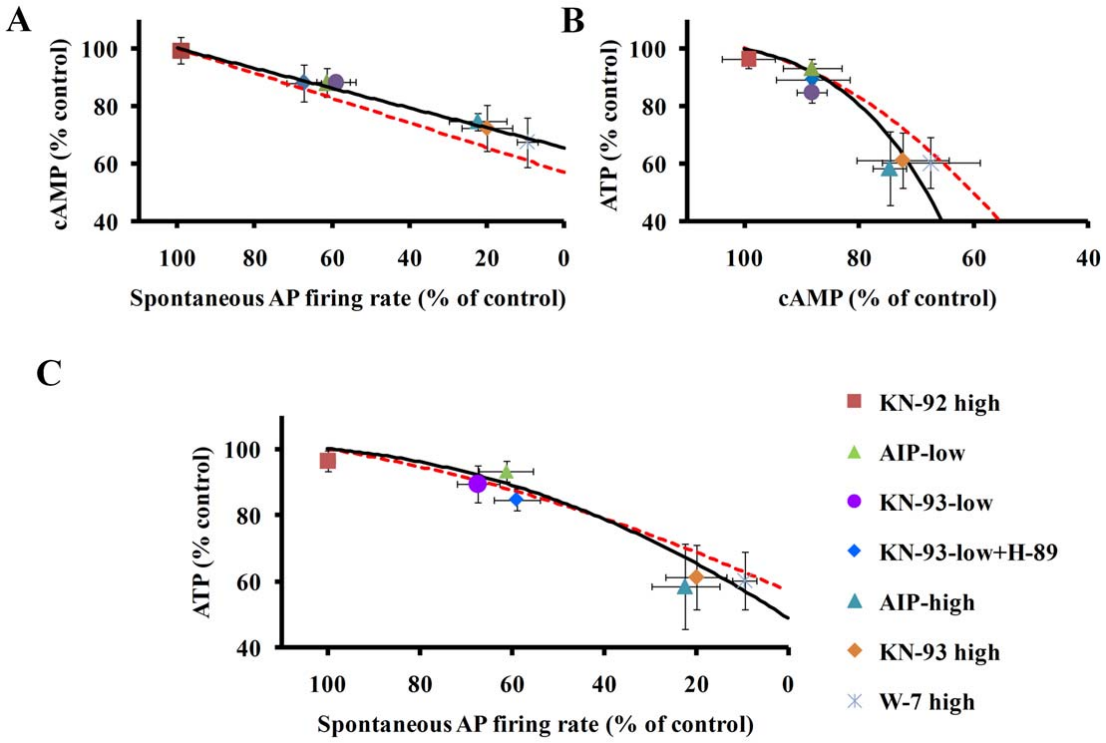
(A) Decreasing CaMKII activity by KN-93 (high-3 µmol/L, low-0.5 µmol/L), AIP (high-10 µmol/L, low-2 µmol/L), or decreasing calmodulin activity by W-7 (high-50 µmol/L, low-20 µmol/L) decreases spontaneous AP firing rate (n = 10 for each drug), cAMP (n = 4 for each drug) and ATP (n = 5 for each drug). The inactive KN-93 analog, KN-92 (3 µmol/L), has no significant effect on these measurements. A decrease in the spontaneous AP firing rate is associated with a decrease in cAMP that is linked to a decrease in ATP (B). Thus, a decrease in spontaneous beating rate is associated with reductions in both cAMP and ATP (B and C), and the reduction in ATP is linked to the reduction in cAMP. CaMKII or calmodulin inhibitor effects measured experimentally are illustrated by the symbols. The solid line is the best fit curve for the CaMKII data. The dashed line is the best fit curve for a battery of drugs that reduce Ca^2+^-cAMP/PKA level measured previously [9].

Fig. 4A shows that graded CaMKII inhibition by KN-93 or AIP in SANC suspensions resultes in graded reductions in cAMP. Graded reductions in cAMP, in response to reduction in CaMKII activity (illustrated by the symbols in Fig 4A), are linearly related to reductions in spontaneous AP firing rate (solid line in Fig. 4A [9]).

Graded reductions in cAMP and AP firing rate effected by KN-93, AIP or W-7 occur concomitantly with graded reductions in ATP (Fig. 4B–C). Note, also in Fig. 4B–C that application of KN-92 neither decreases the spontaneous AP firing rate, nor depletes cAMP or ATP. To clarify the link between the reduction in ATP in response to inhibition of CaMKII signaling and inhibition of cAMP/PKA signaling, we superfused SANC with both KN-93 (0.5 µmol/L) and H-89 (0.5 µmol/L, PKA blocker); at this concentration, each agent reduces the AP firing rate by 40%, i.e., by nearly half of their maximal effects on the AP firing rate. The drug combination did not produce significant additive effects on the reductions in the spontaneous AP firing rate, cAMP or ATP effected by 0.5 µmol/L KN-93 alone (Fig. 4) (but these parameters were significant compared to the control).

It is important to note that blocking AP induced contraction with blebbistatin has no significant effect on AP firing rate or ATP [9] and therefore the reduction in ATP by CaMKII and calmodulin inhibition, per se is not due to blocking SANC contraction. The solid line in Fig. 4 is the best fit curve for the CaMKII data. The dashed line is the best fit curve for a battery of drugs that reduce Ca^2+^-cAMP/PKA level measured previously [9]. This line is similar to the trends achieved with the CaMKII data alone. Moreover, the PKA inhibitor, H-89, is known to decrease PKA dependent phosphorylation mechanisms that resultant in a reduction in cytosolic Ca^2+^ [12]. A reduction in cytosolic Ca^2+^ decreases Ca^2+^-calmodulin cAMP/PKA dependent phosphorylation that further reduces cytosolic Ca^2+^ (Table 3). Furthermore, PKA-dependent phosphorylation of Ca^2+^-calmodulin activated phosphodiesterase 1 inhibits its activity, and reduces cAMP degradation [13].

**Table 3:** Average cells AP induced Ca^2+^ parameters in control (CON) and following introduction of H-89 (0.5 µmol/L; n = 6), *p<0.05 vs. drug control.

	CON	W-7	% Change
**Peak Systolic Ca^2+^** **(nmol/L)**	305±20	297±6^*^	−15±5^*^
**Minimum diastolic** **Ca^2+^ (nmol/L)**	118±20	127±4	−3±4
**Ca^2+^ amplitude** **(systolic-diastolic)** **(nmol/L)**	217±14	170±26^*^	−24±9^*^
**T-P_c_ (ms)**	80±13	82±11	5±9
**T-50_c_ (ms)**	157±27	191±16^*^	12±6
**T-90_c_ (ms)**	282±22	344±15^*^	13±4^*^
**Beats per min**	139±2	93±4^*^	−33±6^*^

**Figure 5:**
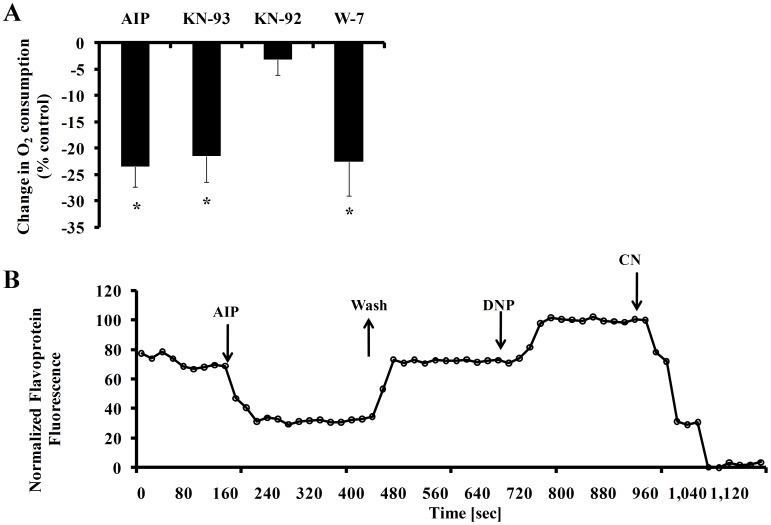
(A) CaMKII inhibition by KN-93 or AIP, or calmodulin inhibition by W-7 decreases O_2_ consumption in SANC suspension (n = 5 for each drug), (B) AIP decreases mitochondrial flavoprotein fluorescence in single SANC (representative example), indicating a net reduction of flavoprotein pool. Washout of AIP reverses this effect. *p<0.05 vs. drug control.

In a coupled ATP-O_2_ system, changes in ATP turnover reflect changes in O_2_ consumption. Application of KN-93, AIP or W-7 decreases O_2_ consumption (Fig. 5A) (Note that KN-92 does not significantly decrease O_2_ consumption). That the reduction in ATP effected by these agents is correlated with the decrease in O_2_ consumption strongly suggests that direct inhibition of Ca^2+^-cAMP/PKA-CaMKII reduces mitochondrial ATP production. Furthermore, application of AIP (10 µmol/L), to reduce CaMKII activity in single SANC decreases the flavoprotein fluorescence (Fig. 5B), an index of the redox potential of the mitochondria. On average AIP significantly reduced the flavoprotein fluorescence by 52±9% from the control value (n = 5). Note that DNP and NaCN were used to calibrate both the flavoprotein fluorescence in each SANC prior to and after AIP application (for further details see method section).

K-ATP channels may exist in SANC and may be activated when ATP level becomes depleted. To explore their potential functional role during basal state AP firing we measured the reduction in ATP in the presence of 3 µM KN-93 and 50 µM pinacidil (a K-ATP channel activator) or 3 µM KN-93 and glibenclamide (a K-ATP channel blocker) and compared it to the reduction in ATP in the presence of 3 µM KN-93 alone. In the presence of KN-93, ATP was reduced by 33±7% (n = 3), in the presence of KN-93 and pinacidil ATP was reduced by 27±3% (n = 3), and in the presence of KN-93 and glibenclamide ATP was reduced by 38±7% (n = 3). Therefore, during CaMKII inhibition, activation of K-ATP channels did not significantly prevent ATP depletion while blocking of K-ATP channels did not produce significant additive effects on ATP reduction.

## Discussion

Basal CaMKII signaling in the absence of β-adrenergic stimulation is crucial for rabbit SANC to generate spontaneous rhythmic AP’s. The most important novel finding of our study is that when ATP demand in SANC is reduced by interfering with CaMKII or calmodulin activity, these cells become depleted of ATP, indicating concurrent reduction in both ATP generation and demand. Thus, CaMKII signaling, a crucial element of normal automaticity in rabbit SANC, is tightly linked to SANC bioenergetics, specifically to both ATP production and utilization. This result is similar to the reduction in ATP by interfering with basal Ca^2+^-cAMP/PKA signaling that has been documented recently [9]. A reduction in ATP level effected by inhibition of CaMKII or calmodulin may lead to a vicious cycle affecting the ATP utilization mechanisms within the cell: SR Ca^2+^ pumping is reduced, myofilament contraction is reduced, Na^+^/K^+^ pump activity is reduced, and may lead to an increase in intracellular Na^+^ and a reduction in Na^+^-Ca^2+^ exchange current that is crucial to prompt spontaneous APs of SANC. However, in permeabilized SANC, in which the ATP level remains intact, both AIP (CaMKII blocker) and PKI (PKA inhibitor) markedly reduced the spontaneous local Ca^2+^ releases and SR Ca^2+^ content [14]. Therefore, a vicious cycle in which the ATP demand affects ATP supply is not the main mechanism by which the CaMKII induces an ATP reduction in rabbit pacemaker cells activity.

Graded CaMKII inhibition by KN-93 or AIP causes graded reductions in cAMP in SANC suspensions (Fig. 2). Therefore, our results showed, for the first time, the existence of mutual entrainment of CaMKII and cAMP in pacemaker cells. When CaMKII activity falls, cAMP activity is reduced too. However, we cannot discriminate the cause-effect relationships of concurrent reductions in CaMKII, spontaneous AP firing rate or cAMP. A reduction in cAMP, however, does not likely derive from the reduction in CaMKII activity, per se, but likely occurs indirectly, from: 1. a decrease in Ca^2+^ influx into the cytosol triggered by each AP (reduced L-type Ca^2+^ channel phosphorylation), and 2. reduced Ca^2+^ release from the SR, related, in part at least, to reduced SR Ca^2+^ pumping [10]. Both mechanisms reduce SR Ca^2+^ loading and Ca^2+^ release, which leads to a reduction in Ca^2+^ activation of AC/cAMP-PKA signaling. Moreover a reduction in the ATP level, as noted, may directly reduce the activity of SR Ca^2+^ pump. Because CaMKII inhibition reduces cAMP, a direct activator of *I_f_*, a reduction in cAMP can decrease the *I_f_* activation, which could also be implicated in the reduction of spontaneous AP firing rate.

We observed that in the presence of AIP the flavoprotein fluorescence is reduced by 52±9% from the control value (Fig. 5). We measured flavoprotein autofluorescence as an index of the redox potential of the mitochondria. It is well known that flavoprotein autofluorescence is reciprocally related to mitochondrial NAD(P)H content. Because NaCN (an electron transport chain inhibitor) is known to reduce flavoprotein fluorescence, and also the cAMP level and spontaneous AP firing rate [9], it may be assumed that CaMKII inhibition reduced the ability of the mitochondria to produce ATP.

K_ATP_ channels are activated with a decrease in intracellular ATP level. The activation of these K_ATP_ channels may play an important role during ischemia [15]. We found here that opening or closing of K-ATP channels plays a small role in ATP –supply to demand budget. Moreover, the relative role of K_ATP_ channels under basal conditions is still controversial. There are no significant differences in the basal AP parameters between WT and Kir6.2 KO SANC mice [16]. In addition, there is reduction in AP firing rate during metabolic inhibition in SANC of Kir6.2 mice. Therefore a decrease in intracellular ATP during metabolic inhibition might result in a decrease in cAMP, thereby decreasing I_Ca,L_, I_f_ and SERCA pump. Depolarization of rabbit SANC membrane occurs not only when CaMKII activity is reduced (as documented in Fig. 1), but also when PKA activity is reduced [12]; note that this effect is reversible), when Ca^2+^ is buffered [3] or when L-type current is blocked [7].

While the spontaneous AP firing rate reduction and ATP depletion demonstrated in the present study (Fig. 2) are effected by a reduction in basal level of CaMKII in rabbit pacemaker cells, conditional knock-out of CaMKII in mice (the predominant CaMKII cardiac isoform [2]) that produces a 40% reduction in CaMKII activity [17] does not alter baseline spontaneous AP firing rate, heart function or survival [18], [19]. However, a decrease in the basal heart rate in CaMKII conditional knock-out mice had previously been reported by the same group [17]. Other reports by the same group [18], [19] in conditional CaMKII knock-out mice show that when no change in baseline spontaneous AP firing rate is observed the PLB phosphorylation also remaines intact. Future experiments are required to determine whether species differences (markedly different spontaneous AP firing rates, markedly differences in ionic channels and Ca^2+^ handling proteins and differences in the relative role of SERCA pump vs. Na^+^-Ca^2+^ exchange) in rabbit SANC or the genetic manipulation in mice account for the differences noted in AP firing rate. These factors might alter the requirement for basal CaMKII activity to maintain basal AP firing rates and compensatory mechanisms (e.g an increase in Ca^2+^ influx) in the knock-out mice.

**Figure 6:**
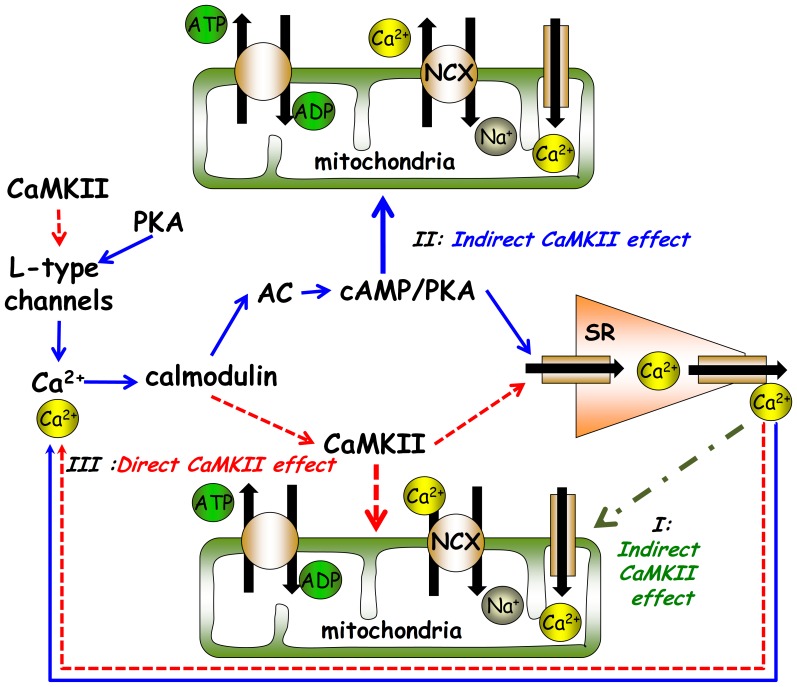
Schematic illustration of the different direct and indirect potential mechanisms of how CaMKII can affect mitochondrial ATP production.

CaMKII activity can affect mitochondrial ATP demand coupling to ATP supply by impacting at least three inter-dependent signaling pathways (Fig. 6): (i) a direct effect of a reduction of cytosolic Ca^2+^; since cytosolic Ca^2+^ is coupled to ATP hydrolysis via mitochondrial Ca^2+^ flux, a decrease in mitochondrial Ca^2+^ in response to CaMKII inhibition can decrease the activity of mitochondrial enzymes involved in ATP production. We have recently shown, however, that directly disabling mitochondrial Ca^2+^ influx or efflux in SANC does not affect the ATP level [9]. (ii) An indirect effect via reduction in cAMP/PKA phosphorylation signaling, as a decrease in SR Ca^2+^ loading and release leads to a reduction in AC-cAMP/PKA-dependent phosphorylation (Fig. 4 B–C) of several mitochondrial proteins [9]. (iii) a direct Ca^2+^-calmodulin dependent phosphorylation of mitochondrial proteins; phosphorylation of both inner or outer mitochondrial membrane proteins (i.e., ATP synthase [20]) has been documented. It has become appreciated over the last several years that protein phosphorylation within the cardiac mitochondrial matrix and respiratory complexes is extensive. To date several mitochondrial molecules, including the voltage dependent anion channel, cytochrome C, and complexs I and V have been found to be phosphorylated by PKA [21]. The specific mitochondrial targets that can be phosphorylated by CaMKII are not well explored. However, phoshorylation of complex V by CaMKII has been demonstrated [20]. Thus, changes in the concentrations of protein phosphorylation in the cytosol may affect mitochondrial ATP production via the exchange of cAMP/PKA and/or CaMKII to the mitochondria. Therefore, it is not known whether a reduction in CaMKII reduces ATP via an indirect effect, i.e., a reduction in cAMP/PKA phosphorylation signaling (mechanism ii), or via a direct Ca^2+^-calmodulin activation-dependent phosphorylation of mitochondrial proteins (mechanism iii). However, because both mitochondria and activated autophosphorylated CaMKII predominantly localize close to the SANC sarcolemmal membrane [7], direct phosphorylation of several mitochondrial proteins might be expected within this area. Future studies to directly measure in real time the response to changes in cAMP, CaMKII activity and ATP signals within intracellular micro-domains to CaMKII inhibition in SANC are required to determine the kinetics and co-localization of PKA-dependent and CaMKII-dependent phosphorylation of mitochondrial complex subunit proteins.

Two different indirect mitochondrial mechanisms can also play a role in matching ATP supply to demand in the heart: (i) mitochondrial respiration and ATP synthesis in the heart are coupled to creatine and synthesis of phosphocreatine (PCr) by the mitochondrial creatine kinase (CK), a key component of the cellular system of creatine kinase CK. In the ventricular myocytes CK is localized to myofibrils at M-line and I-band of sarcomeres and subsarcolemmal space and serve as a control mechanism to match ATP supply to demand [22]. However, in SANC myofilament density is relatively low [23] and application of blebbistatin, which completely blocks contraction, neither decreases the spontaneous AP firing rate, nor cytosolic Ca^2+^ nor the ATP level [9], [24]. These results imply that myofilaments are not the major consumer of ATP in SANC as in ventricular myocytes. Future experiments are necessary to explore the relative role of CK in pacemaker cells. (ii) The ATP can also be supplied by cytosolic glycolysis (for review see Saks et al., Int J Mol Sci 2008). However, we have previously demonstrated that glycolysis does not support normal basal ATP production in pacemaker cells [9]. Moreover, the concomitant reductions oxygen consumption and flavoprotein fluorescence during CaMKII inhibition suggests that the mitochondria are probably the target of the decrease in CaMKII and/or cAMP/PKA.

### Limitations

It has been reported that KN-93 may affect I_Ca_ [25] and a variety of voltage-gated K^+^ currents [26]. To verify the specificity of KN-93 we used two approaches. We used KN-92, an inactive KN-93 analog. KN-92 has no significant effect on spontaneous AP firing rate, cAMP, ATP and O_2_ consumption measurements. But because KN-92 does not share the CaMKII-independent L-type Ca^2+^ channel antagonist actions of KN-93 (for review cf. [27]), we also employed a specific CaMKII inhibitory peptide, AIP that is not known to affect ion channels [28]. Our results demonstrate that AIP has similar effects as KN-93 on spontaneous AP firing rate, cAMP, ATP and O_2_ consumption. Thus, KN-93 and AIP appear to be legitimate pharmacological tools to explore CaMKII inhibition effects. W-7 may also inhibit Ca^2+^ pumping by SERCA pump, but it is the least potent inhibitor of all known calmodulin inhibitors (Ic_50_ = 125 µM, less than 20% decrease in SERCA activity at 50 µM) [29].

## Materials and Methods

In the presence and absence of specific CaMKII inhibitors, we measured the spontaneous AP firing rate from cell contraction or electrophysiology recordings, intracellular Ca^2+^, and flavoprotein autofluorescence in single isolated rabbit SANC and ATP, cAMP and O_2_ consumption in SANC suspensions.

### Single SANC Isolation

Single, spontaneously beating, spindle-shaped SANC were isolated from New Zealand White rabbit hearts as previously described [30]. The study conformed to the Guide for the Care and Use of Laboratory Animals published by the US National Institutes of Health. The experiment protocols were approved by the Animal Care and Use Committee of the National Institutes of Health (protocol # 034 LCS 2013). The rabbits weighed 1.8–2.5 kg and were deeply anaesthetized with sodium pentobarbital (50–90 mg/kg) injected to the central ear vein. The adequacy of anesthesia was monitored until reflexes to ear pinch and jaw tone were lost.

### Cell Contraction and Electrophysiological Recordings

The cell spontaneous AP firing rate from contraction or electrophysiological recordings were measured in Tyrode solution at 35±0.5°C containing (in mmol/L): 140 NaCl, 5.4 KCl, 2 MgCl_2_, 5 HEPES, 1.8 CaCl_2_, and 5.5 Glucose, pH 7.4 with NaOH. Spontaneous APs were measured via perforated patch-clamp technique with 35–50 µmol/L β-escin (Sigma) added to the pipette solution that contained (in mmol/L): 120 K-gluconate, 2.5 NaCl, 2.5 MgATP, 2.5 Na_2_ATP, 5 HEPES and 20 KCl, and titrated to pH 7.2 with KOH. APs were recorded using an Axopatch-200 B patch-clamp amplifier (Axon Instruments, Foster City, CA). To quantify the rate of spontaneous AP firing contraction, in cells in which APs were not measured directly, cells were imaged with an LSM-510 inverted confocal microscope using a 63x/1.4 N.A. oil immersion lens (Carl Zeiss), and 633 nm He-Ne laser excitation. The bath solution and the temperature were the same as in spontaneous AP recording experiments. Transmitted optics line-scan images (using 512x1 pixels at 21.5 pixel/µm and 0.8 ms/line) were recorded with a scan line oriented along the short axis of the cell to quantify the SANC contraction. The spontaneous AP firing rate was calculated as the time between the successive contraction periods. For AP and contraction recordings cells from at least 3 rabbits were used for the study of each drug.

### Ca^2+^ Measurements

Intracellular Ca^2+^ was measured via calibrated Indo-1 fluorescence in Tyrode solution (described above) at 35±0.5°C as described before [31]. SANC were placed in a chamber on an inverted fluorescence microscope stage (Zeiss IM-35), were loaded with 14 µmol/L Indo-1 AM (Molecular Probes) for 15 min at room temperature, and were subsequently superfused with Tyrode’s solution (see above) for 20 min to remove excess indicator. To allow full de-esterification, the bath temperature was slowly increased to 35±0.5°C. The Indo-1 was excited by a xenon arc at 350±5 nm and collected at 410 and 485 nm. The apparatus to detect Indo-1 fluorescence described previously [32], except that a 63x/1.4 N.A. oil UV fluorglycerin-immersion objective (Zeiss) was used. Indo fluorescence was corrected for background and autofluorescence determined in every experiment by subtracting averaged signals from cells not loaded with Indo-1 (n = 5). Note that incubation of SANC with indo-1 slightly decreased the AP firing rate (∼10%). [Ca^2+^]_i_ was calculated according to the equation [Ca^2+^]_i_ = βxK_d_x(R-R_min_)/(R_max_-R), using a K_d_ of 844 nM. The average R_min_ (minimal ratio), R_max_ (maximal ratio), and β (the ratio of maximal and minimal I_485_) for the fluorescence system were determined by sequential exposure of SANC to a high potassium, zero- Ca^2+^ solution (in mM: 132 KCl, 10 Hepes, 2 MgCl_2_, pH 7.2 with KOH) containing metabolic inhibitors (10 mM 2-deoxyglucose and 100 µM 2,4-dinitrophenol) (2), the same solution with 1 mM EGTA and 20 µM ionmycin (for R_min_). Measurements were taken when the fluorescence at both wavelengths reached stable values, and (3) high Ca^2+^ Tyrode’s solution (5 mM Ca^2+^ substitutes for EGTA) was used for determining R_max_. Average R_max_, R_min_ and β were 1.9±0.16, 0.9±0.01, and 2.1±0.6, respectively (n = 10). All these measurements (step 1 to 3) were performed in Tyrode solution (described above) at 35±0.5°C. Note that this calibration method has two limitations: 1) a change in pH from the calibrated solution (pH = 7.4 in bath solution compared to 7.2 during calibration), 2) imprecision of the K_d_ of Indo-1. For Ca^2+^ recordings cells from at least 5 rabbits for each drug application were used.

### Flavoprotein Autofluorescence Recordings

The endogenous autofluorescence of mitochondrial flavoproteins was imaged in Tyrode solution (see above) at 35±0.5°C by a LSM510 confocal microscope (see above) using a 40x/1.3 N.A. oil immersion lens, as previously described [9]. Flavoprotein autofluorescence was recorded (20 s/frame) using a 488 nm laser. The flavoprotein fluorescence (an indicator of matrix redox state) was expressed as percent change of control that was normalized to the maximum flavoprotein fluorescence (using 100 µmol/L 2,4-dinitrophenol (DNP)) and minimal flavoprotein fluorescence (using 4 mmol/L NaCN).

### Experimental Protocol for Cell Suspensions

Because the number of healthy and functional cells in cell suspensions varies from preparation to preparation (from 20 to 30%), comparability among responses to CaMKII or calmodulin inhibitors in different suspensions was insured by measuring ATP, cAMP and O_2_ consumption in aliquots of a given suspension containing an equal amount of protein and viable cells. Therefore, to compare results of different suspensions studied on different days, drug effects in aliquots from all suspensions were expressed as a % of their respective controls. Lack of significant contamination by atrial cells was verified by classifying morphology and counting cells under the microscope. Finally, to insure the validity of comparison between ATP, cAMP and spontaneous beating rate, the beating rates were measured in single SANC extracted from the same suspension in which ATP, cAMP and O_2_ were measured.

### O_2_ Consumption Measurements

Oxygen consumption was measured in cell suspensions of spontaneously beating SANC using Clark-type electrodes (MT200, Strathkelvin Instruments Ltd.). SANC suspensions were centrifuged at 100 g for 5 min, the supernatant removed, and SANC were incubated in fresh Tyrode solution (as above) that was not oxygenated prior and during these experiments. The cell suspension was divided equally into 2 aliquots: the first aliquot for specific CaMKII or calmodulin inhibition protocols, and the second to serve as a control. Cell suspensions were stirred gently at 36°C, and measurements were acquired for 2 min under control conditions, and 3 min following application of CaMKII or calmodulin inhibitors. Measurements in the control were made for 5 min, and reported at the end of 5 min. Oxygen consumption in the presence of drug was normalized to the control level for each experiment (to normalize number of cells, % of viable cells and oxygenation conditions that vary from one isolation to the other). To guarantee that cells within the suspension were functioning normally and reacting to the drug application, spontaneous beating rates, prior and after specific pharmacological probes used to probe O_2_ consumption, were measured on single cells from the same cell suspension.

### ATP Measurements

The cell suspension (incubated in fresh Tyrode solution (as above)) was divided equally into 6 aliquots: the first aliquot was treated with a CaMKII or calmodulin inhibitor, the second with 100 µmol/L DNP for 15 min, in order to uncouple SANC energetics and to decrease ATP level; the third and the fourth were used as controls, and the other two remaining aliquots were used to measure cAMP (see above). After a 5-min incubation period at 36°C (cells were incubated in an oxygen measurement chamber, see above) the reactions were stopped by adding to the cell suspensions, in a 1∶10 ratio, TRIS-lysis buffer that was heated in a boiling water bath. The TRIS-lysis buffer contained (in mmol/L) 100 TRIS and 4 EDTA, with pH adjusted to 7.75 with HCl. The extracts were heated in boiling water for 3-min and centrifuged at 12,755 g for 2-min at room temperature. Total protein concentration was determined by a BCA™ Protein Assay (Pierce), and the number of viable (spontaneously contracting) cells was determined from the original cell suspension before the reactions were stopped. The ATP concentration was determined by a bioluminescence assay kit, HS II (Roche), and normalized to the cell ATP level after 15-min incubation with 2,4-dinitrophenol, as previously described [9]. To guarantee that the cells within the suspension in which ATP was measured were functioning and reacting to the specific pharmacological probes, spontaneous beating rates were measured on single cells from the same cell suspension.

### cAMP Measurements

Two groups of cell suspensions (control and drug) were pretreated with 100 µmol/L IBMX prior to drug applications. The reaction was stopped by adding the cell suspensions, in 1∶1 ratio, to heated (in a boiling water bath) TRIS-lysis buffer (see above). The extracts were heated in boiling water bath for 5 min and centrifuged at 12,755 g for 2 min at room temperature. The supernatants were collected in fresh tubes and cAMP levels were measured by a LANCE cAMP detection kit (PerkinElmer) and expressed as percentage of the drug-free, control group.

### Drugs

CaMKII inhibitor KN-93, CaMKII inactive analog KN-92 and a specific cell permeable PKA inhibitor peptide autocamtide-2 Related Inhibitory Peptide (AIP), Myristoylated were obtained from EMD Chemicals; 3-Isobutyl-1-methlxanthine (IBMX), N-(6-Aminohexyl)-5-chloro-1-naphthalensulfonamide hydrochloride (W-7), PKA inhibitor (H-89), NaCN and 2,4-dinitrophenol (DNP)) were obtained from Sigma.

### Statistical Analysis

Data are presented as mean±SEM. T-test was employed to compare the drug effect in paired samples (Ca^2+^ measurements). ANOVA test was employed to compare paired samples with different drug concentration. *P<*0.05 was taken to indicate statistical significance.
